# CXCR4 Inhibition Induces Tumor Necrosis by Selectively Targeting the Proliferating Blood Vessels in Oral Squamous Cell Carcinoma

**DOI:** 10.7150/jca.115847

**Published:** 2025-09-22

**Authors:** Yamin Soe, Hotaka Kawai, Htoo Shwe Eain, Saori Yoshida, May Wathone Oo, Zin Zin Min, Kiyofumi Takabatake, Keisuke Nakano, Hitoshi Nagatsuka

**Affiliations:** 1 Department of Oral Pathology and Medicine, Graduate School of Medicine, Dentistry and Pharmaceutical Science, Okayama University, Okayama, 7008558, Japan.; 2 Department of Surgery, Columbia University Irving Medical Center, New York, NY 10032, United States.; 3 Preliminary Examination Room, Okayama University Hospital, Okayama, 7008558, Japan.; 4 Department of Pathophysiology and Drug Discovery, Graduate School of Medicine, Dentistry and Pharmaceutical Science, Okayama University, Okayama, 7008558, Japan.

**Keywords:** CXCR4, tumor angiogenesis, chemokine receptors, tumor microenvironment, oral squamous cell carcinoma (OSCC), AMD3100

## Abstract

The C-X-C chemokine receptor type 4 (CXCR4) is a G protein-coupled transmembrane receptor that contributes to tumor growth and angiogenesis. While prior studies have primarily focused on CXCR4 expression in cancer cells and its role in metastasis, a few have examined its involvement in tumor-associated vasculature. In this study, we reported for the first time that CXCR4 expression within the tumor vasculature is significantly associated with higher pathological grades of human oral squamous cell carcinoma (OSCC) (p<0.03). A previous study reported that inhibiting CXCR4 with AMD3100 induces tumor cell death and enhances the efficacy of the chemotherapeutic agent cisplatin. These findings suggest that CXCR4 is an important target for cancer treatment. However, the tumor vascular system is known to be heterogeneous within the tumor microenvironment (TME), which may influence the treatment outcomes. Therefore, this study aimed to explore the effect of CXCR4 antagonism on various blood vessels present within the oral squamous cell carcinoma (OSCC) tumor stroma. Although the efficiency of AMD3100 was not significant in MOC cancer cells, necrosis was induced in the TME when applied to a poorly differentiated OSCC model, highlighting the role of the TME. Notably, CXCR4 is found to be highly overlapped with CD105^+^ angiogenic tumor vessels among various vascular markers. Treatment with AMD3100 leads to a marked reduction in the CD105^+^ vessels and impairs the maturation of tumor micro-vessels, explaining the cause of observed necrosis. Thus, CXCR4 serves as a promising biomarker in OSCC, and its inhibition with AMD3100 offers the therapeutic potential, particularly in cases with advanced pathological grades.

## Background

Oral squamous cell carcinoma (OSCC) is a solid neoplasm and is the most common type of head and neck cancer^1^,^2^. It arises from the mucosal epithelium of the oral cavity and is histologically characterized by cellular atypia and squamous differentiation^2^. Well-differentiated OSCCs are low-grade tumors with cells resembling normal squamous cells, leading to slower growth and spread. In contrast, poorly differentiated OSCCs are associated with abnormal cellular features, disorganized appearance, faster and aggressive growth, and higher metastasis, which often result in poor clinical outcomes^3^,^4^. Differences in histological differentiation are crucial for OSCC prognosis^3^-^5^. Moreover, these OSCC subtypes exhibit different patterns of tumor vascularity and treatment responses. Well-differentiated OSCC tend to have more stable and lower vascular density than poorly differentiated tumors, which exhibit higher vascular density with an immature and disorganized vascular architecture owing to their aggressive nature^6^. Vascular density is correlated with tumor aggressiveness and prognosis, making it a crucial factor in treatment planning^7^,^8^. The tumor vasculature, which is characterized by diverse vessel characteristics and levels of maturity, influences the effectiveness of anti-cancer treatments^9^,^10^. The blood vessels supplying the tumor exhibit significant differences from their normal counterparts and display high heterogeneity in organization, structure, and function^6^,^11^. Angiogenesis is the process of forming new blood vessels from existing ones and is characterized by abnormal blood vessel growth in the tumor microenvironment (TME)^12^. It ensures the delivery of oxygen and nutrients to primary cancer cells and is essential for tumor growth and progression^12^,^13^. Therefore, many anti-vascular treatments have emerged as therapeutic strategies to disrupt angiogenesis. Tumor angiogenesis is regulated by a complex interplay between various factors in the TME, including endothelial cells, cytokines, growth factors, and stromal cells^13^-^15^. Endothelial cells are specialized cells that line the inner surface of tumor blood vessels. Vascular endothelial cells are highly diverse and can be categorized into subgroups based on their unique gene expression, morphology, and functions^16^. They express receptors for angiogenic and growth factors, which serve as signalling molecules to promote or inhibit angiogenesis^17^. Targeted treatment based on these molecules, particularly the vascular endothelial growth factor (VEGF), is a promising strategy for anti-tumor therapy. However, not all patients show effectiveness in treatments that inhibit VEGF, highlighting the need for reliable biomarkers to predict the response to therapy^18^,^19^.

C-X-C chemokine receptor type 4 (CXCR4) is a G protein-coupled transmembrane receptor initially identified in leukocytes. Along with its ligand, stromal-derived factor-1 (SDF-1), CXCR4 plays an essential role in homing hematopoietic cells to the sites of inflammation^20^. It is also expressed in over 23 different types of cancers and is known to regulate pathways involved in tumor progression, invasion, metastasis, and angiogenesis^21^-^24^. Therefore, a CXCR4 receptor antagonist, AMD3100, is gaining interest as an anti-cancer agent by disrupting the interaction between cancer and TME^25^,^26^. Although previous studies have explored CXCR4 expression in cancer cells and its role in promoting metastasis, comparatively few have addressed its function within tumor-associated vasculature, particularly in relation to angiogenesis and vascular remodeling. One prior study reported that CXCR4 expression is observed in the stromal endothelial cells of OSCC and that CXCR4 inhibition with AMD3100 induces tumor necrosis^27^. However, tumor vasculature is heterogeneous, and the exact mechanism by which CXCR4 inhibition induces tumor necrosis remains unclear. In this study, we investigated the effects of CXCR4 antagonism on diverse blood vessels within the OSCC tumor stroma.

We discovered that CXCR4-expressing tumor vessels are significantly correlated with the histological differentiation of human OSCC microarray. Furthermore, our data indicate that although CXCR4 co-localizes with diverse vascular structures in the stroma of the orthotopic OSCC mouse model, the inhibition of CXCR4 selectively affects the active angiogenic marker CD105^+^ endothelial cells in the poorly differentiated tumor stroma and diminishes the maturation of CD105^+^ vessels, ultimately resulting in tumor necrosis.

## Materials and Methods

### Cell line and cell culture

Mouse oral cancer cell lines, MOC1 (KER-EWL001-FP) and MOC2 (KER-EWL001-FP) were purchased from Kerafast cell bank and cultured according to the protocol provided by the laboratory of R. Uppaluri at Washington University (St. Louis, MO, USA). The cell lines used in this study were tested for infectious disease PCR, interspecies contamination test, and mouse short tandem repeat profile by the supplier. Cells were cultured in 2:1 medium of Iscove's Modified Dulbecco's Medium (IMDM) (12440046, Gibco, Grand Island, NY, USA) and Ham's Nutrient Mixture (11765054, Gibco) with additional 5% FBS (Gibco), 1% antimycotic- antibiotic solution (Anti-Anti) (15240062, Gibco), 5 mg/ml insulin (I6634, Sigma-Aldrich, St.Louis, MO, USA), 40 µg/ml Hydrocortisone (H0135, Sigma Aldrich) and 5 µg EGF (585508, Biolegend, San Diego, CA, USA) at 37 °C in a humidifying incubator with 5% CO_2_.

### Reagent

Ten milligrams of AMD3100 (Plerixafor) (CA13074-10, ADQ Adooq BioScience LL) were purchased and dissolved in 20 ml of saline. The reagent is then deliquated and stored at 4 °C for usage. The deliquated vials were wrapped in aluminum foil to shield them from light and prevent degradation.

### MTS assay

MOC1 and MOC2 cells (1.5 x 10^3^ cells per well) were seated separately in 96-well plates with 200 µl of medium in each well. Two concentrations of AMD3100 (1 µg/ml, 2 µg/ml) were used. Following the AMD3100 incubation period of 0 h, 24 h, and 48 h in a 37 °C incubator with 5% CO_2_, 20 µl of Cell Titer 96 Aqueous One Solution Cell Proliferation Assay (G3580, Promega, Madison, WI, USA) was added to each well and further incubated for 2 h before measurement. The absorbance of each well was measured at a wavelength of 490 nm using an enzyme-linked immunosorbent assay reader (SH1000lab). The experiment was repeated thrice.

### Transwell migration

MOC1 (1 × 10^5^ cells) and MOC2 (5 × 10^5^ cells) were seeded in 24-well plates with 300 µl of IMDM medium in each well without FBS at the upper chamber with a pore size 8 µm. The lower chamber contains 750 µl of IMDM with 10% FBS. Two AMD3100 concentrations, 1 µg/ml and 2 µg/ml, are added to the upper chamber and incubated for 24-48 hours at 37 °C with 5% CO_2_. After incubation, the migrated cells were stained using Giemsa staining with Diff-Quik solution (16920 Sysmex), and the non-migrated cells from the upper chamber were removed with cotton swabs. Images of the migrated cells were taken using a microscope BX53 (Olympus), and the cells were counted using ImageJ 1.52a software. The experiment was repeated thrice.

### Animal model

Wild-type C57BL/6J female mice (8 weeks old) were purchased from The Jackson Laboratory Japan (Yokohama, Japan). All the mice were housed in a standard animal facility in a pathogen-free environment.

### Orthotopic tumor transplantation and AMD3100 administration

MOC1 at 2 × 10^6^ cells/33% Matrigel (Corning) with HBSS (Invitrogen, Thermo Fisher Scientific, Waltham, MA, USA) and MOC2 at 3 × 10^4^ cells/HBSS were transplanted into the right buccal mucosa of C57BL/6J mice^28^. The MOC2 transplanted mice (n=5) were sacrificed after three weeks, while the MOC1 transplanted mice (n=5) were sacrificed after four weeks. For drug treatment, MOC1 and MOC2 were transplanted again in the same manner, and a daily injection of AMD3100 (2.5 mg/kg, n=5) and saline as control (n=4) were given intraperitoneally for 14 consecutive days before sacrificing at four weeks and three weeks for MOC1 and MOC2, respectively. Humane endpoints were defined as a condition of the mouse that worsened after drug administration, with rapid progression of pain, decreased food intake, and reduced mobility within 3 days, and the pain was deemed unbearable. In this case, the mouse was euthanized by cervical dislocation or carbon dioxide gas.

### Tissue processing and histological analysis

The mice were euthanized with an overdose of isoflurane inhalation (5% dosage for a prolonged period) and verification of death by cervical dislocation. After the mice were sacrificed, the tumor tissues were excised and fixed with a 4% paraformaldehyde solution for 36-48 h. Bone tissues were decalcified in 10% EDTA at 4 °C for 10-14 days until the tissues were softened enough for cutting. The tumors were sectioned at the largest cross-sectional area. The tissue samples were dehydrated starting from 70% ethanol solution and gradually increasing to 100% alcohol. Xylene was used as the clearing agent before the samples were embedded in paraffin. Paraffin sections were excised 3-4 µm thick cross-sections and then prepared for staining methods. The sections were stained with hematoxylin and eosin (H&E) and analyzed using a BX53 light microscope (Olympus). For serial immunohistochemical (IHC) analysis, each tumor tissue was excised consecutively and mounted on the glass slides in serial order for analysis.

### Microarray tissue staining

Commercially available high density oral cavity squamous cell carcinoma and normal tissue array containing 60 cases (28 from tongue tissue, 15 from gingiva, 5 from oral cavity, 3 from lower jaw, 2 from soft palate, 2 from maxillary sinus, 2 from cavioris bucca, 2 from lip, 1 from palate), 9 tongue tissues (OR208a) was purchased from TissueArray.Com LLC (Derwood, MD20855, United States), which provides clinicopathological data such as TMN staging based on AJCC classification. One case was excluded due to tissue detachment. The slide was baked for 1 hour at 60 °C prior to staining.

### Immunohistochemistry (IHC) staining

Peroxide elimination was performed by blocking with hydrogen peroxide/methanol solution for 30 min. Antigen retrieval was performed according to the manufacturer's instructions, and the samples were allowed to cool for at least 2 h. After washing with TBS, protein blocking (Vector Laboratories, Newark, CA, USA) was performed for 15 min at room temperature (RT). Then, the primary antibodies detailed in Supplementary Table S1 are incubated at 4 °C overnight. The slides were incubated with secondary antibodies for 30 min at RT. Signals were enhanced with an avidin-biotin complex, and staining was performed with diaminobenzidine (DAB). The DAB incubation period was determined using the pilot slides. Mayer's hematoxylin (Millipore Sigma) was used as a counterstain. The slides were then dehydrated with increasing alcohol concentrations, and xylene was added. The staining results were visualized, and images were taken with an optical microscope (BX53, Olympus). Positive vessel structures were counted in five random hot spots of each tumor using ImageJ 1.52a software.

### Double immunofluorescence (IF) staining

Following antigen retrieval, the samples were blocked with a blocking buffer (DS Pharma Biomedical) for 20 min at RT. Then the slides were incubated with primary antibodies detailed in Supplementary Table SI at 4 °C overnight. After washing with TBS, the secondary antibody was applied at a dilution of 1:1000 for 1 hour at RT. The secondary antibodies used are Alexa Flour 568 anti-rabbit IgG (A-10042, AbCam, Tokyo, Japan) for CXCR4 and α-SMA, Alexa Flour 594 anti-rat IgG (A-21209, AbCam, Tokyo, Japan) for CD34, and Alexa Flour 488 anti-goat IgG (A-11055, AbCam, Tokyo, Japan) for CD105. The sections were stained with the nuclear stain DAPI (D523, Dojindo Laboratories) and then visualized using an All-in-one BZ x700 fluorescence microscope (Keyence).

### Quantification and statistical analysis

We randomly captured five images per mouse (× 400 magnification) of tumor sections. Tumor dimensions (length × width) were measured with a caliper starting from the drug injection day, every other day during the tumor growth period. Necrosis area measurement and positive vessel counting were performed using ImageJ 1.52a. All in vitro experiments were independently repeated at least three times with consistent results, showing representative results. All statistical analyses were performed using GraphPad Prism 10.0.1. Two-tailed student t-test for independent samples with equal variances was used to compare the two groups. One-way ANOVA followed by Tukey's multiple-comparison post hoc test was used to compare more than three groups. The difference was considered significant at p values less than 0.05, and all data are presented as the means ± standard deviation (SD). The chi-square test was used to analyze the significant differences between CXCR4-expressing tumor vessels and clinico-pathological parameters in human OSCC tissues. We performed a Z-score analysis, and no data points exceeded the commonly used threshold (±3.0). Therefore, no outliers were identified, and all data points were retained for analysis.

## Results

### CXCR4 expression in OSCC microarray and its relation to tumor differentiation and prognosis

**Figure 1:**
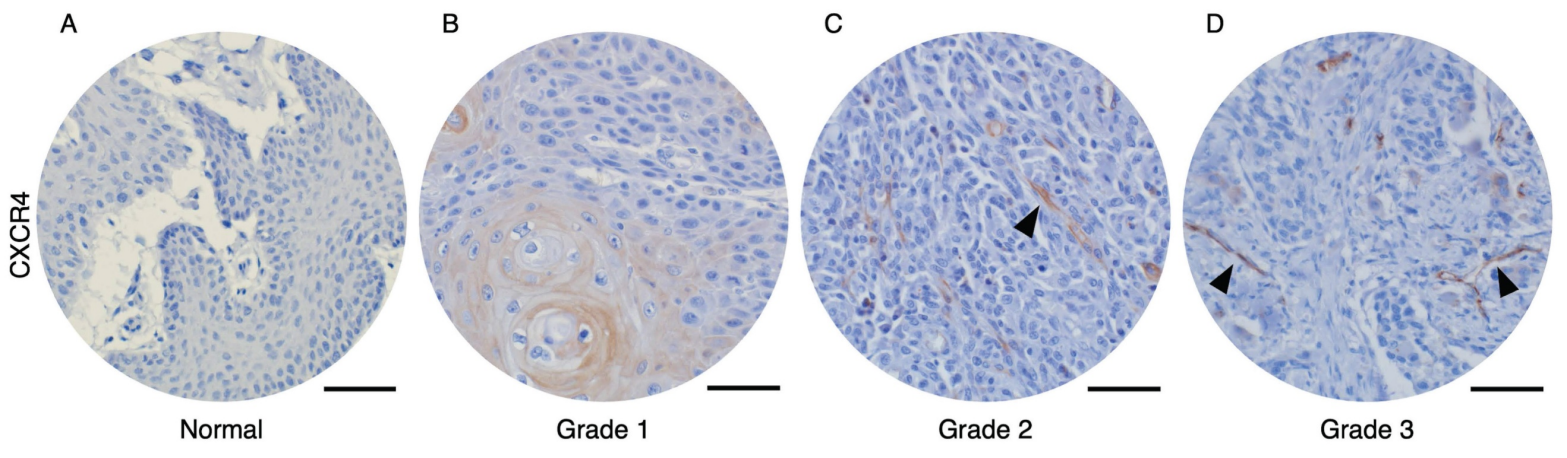
Immunohistochemical analysis of OSCC microarray tissue revealed that CXCR4 expression is not detected on normal oral mucosa (A) and Grade 1 OSCC tissues (B). In contrast, CXCR4^+^ vascular structures were observed in Grade 2 (C) and Grade 3 (D) OSCC specimens. The arrows indicated the CXCR4-expressing tumor vessels. Scale bar: 50 µm.

To analyze the correlation between CXCR4-expressed tumor vessels and clinical parameters, we stained the CXCR4 in human oral squamous cell carcinoma (OSCC) and normal tissue microarrays. Immunohistochemical analysis of human OSCC tissue microarrays revealed that tumor blood vessels expressing CXCR4 were not observed in normal oral mucosa (Figure 1A) or Grade 1 OSCC tissues (Figure 1B). In contrast, distinct CXCR4^+^ vessel-like structures were detected in Grade 2 and Grade 3 OSCC tissues. Quantitative analysis, summarized in Table 1, demonstrates a significant correlation between the presence of CXCR4^+^ vessels and tumor pathological differentiation. These results imply that CXCR4 expression in the vasculature is associated with tumor aggressiveness and its potential role in promoting tumor angiogenesis and progression.

### MOC1 tumor exhibit well-differentiated features, while MOC2 tumor exhibit poorly differentiated features

**Figure 2:**
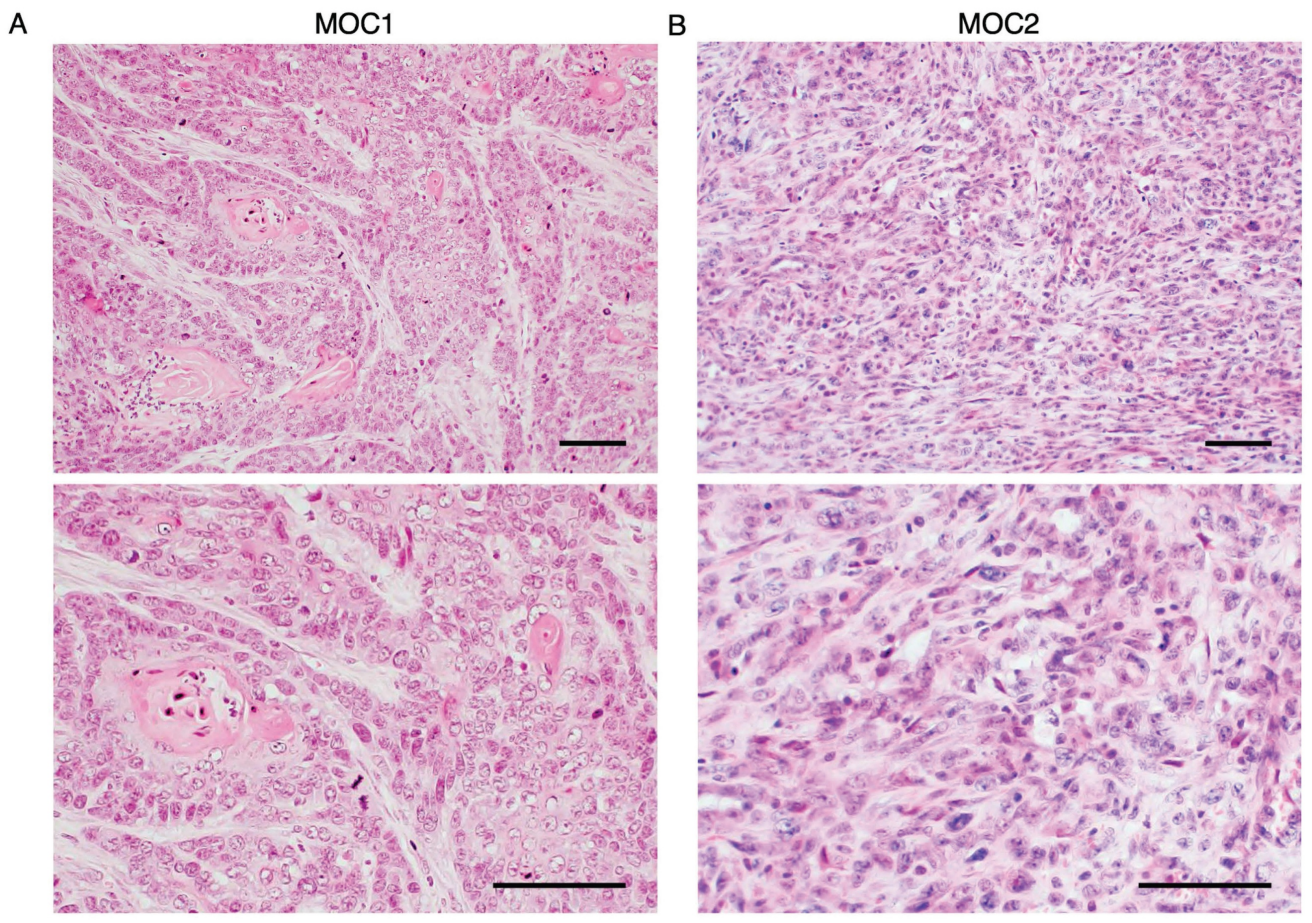
Histological analysis of MOC1 and MOC2 tumor. H&E images of (A) MOC1 exhibiting a well-differentiated phenotype with nests of tumor cells and keratinisation. (B) The stroma and tumor cells lacked resemblance to normal squamous cells and were intermingled without clear boundaries, indicating poorly differentiated characteristics of MOC2. Scale bar: 50 µm.

We analyzed the hematoxylin and eosin (H&E) staining of MOC1 and MOC2 transplanted tumor tissues to understand the variants of the two transplanted MOC cell lines. MOC1 tumors show nests of tumor cells with keratinisation. The tumor nest had a clear boundary from the stromal area, which is characteristic of a well-differentiated variant (Figure 2A). MOC2 tumors are comprised of tumor cells with higher nuclear-cytoplasmic ratios, and pleomorphic nuclei. The stromal area and tumor cells were intermingled with no clear boundaries. Therefore, MOC2 showed characteristics of poorly differentiated variants (Figure 2B).

### Distinct characteristics of CD34^+^ and CD105^+^ vessel structures were observed in the stroma of low-angiogenic MOC1 and high-angiogenic MOC2 tumors

**Figure 3:**
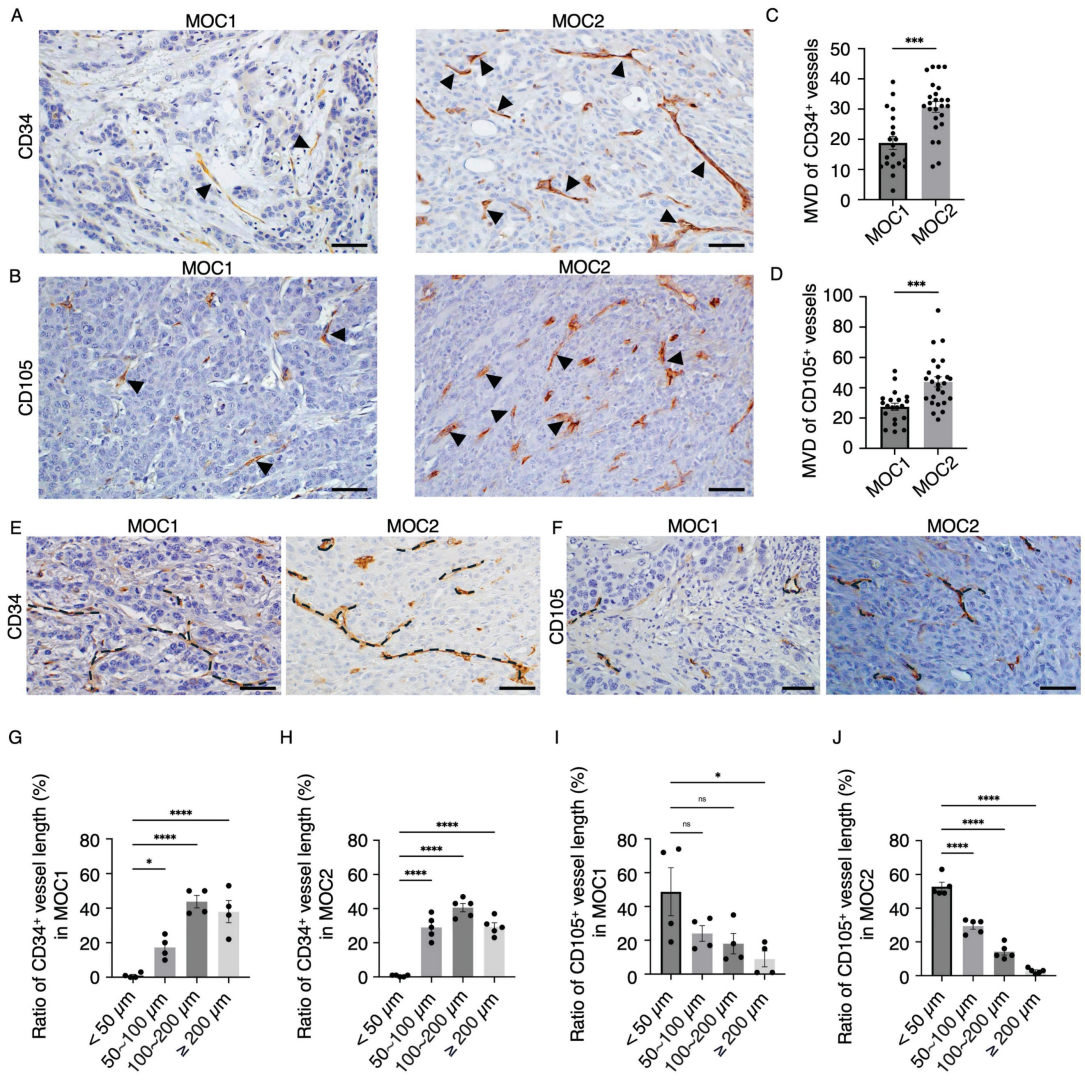
MOC1 and MOC2 tumor vessel landscape. IHC images of (A) CD34^+^ vessel-like structures in MOC1 & MOC2. IHC images of (B) CD105^+^ vessel-like structures in MOC1 & MOC2. Arrows indicate positive vessel-like structures. Scale bar: 50 µm. The number of (C) CD34^+^ and (D) CD105^+^ vessel-like structures is significantly higher in MOC2, indicating greater angiogenic activity. (E) CD34^+^ vessel structures are various-sized vessels, while (F) CD105^+^ vessel structures are short and irregular. Dotted lines represent the vessel structures. Scale bar: 50µm. The measurement of CD34^+^ vessel length ratio shows that various lengths ranging from <50-200≤ µm vessel structures are observed in both (G) MOC1 and (H) MOC2. The vessel length ratio for CD105^+^ indicates a high number of vessel structures smaller than 100 µm in (I) MOC1 and (J) MOC2. All data is presented as mean ± SD. Statistical analysis was done using the student's t-test for comparison of two groups and by one-way ANOVA, followed by Tukey's multiple-comparison post hoc test. *P<0.1, ***P<0.001, ****P<0.0001.

To visualize the vessel landscape of MOC1 and MOC2 tumors, the endothelial cell markers CD34 and CD105 were stained separately because of their heterogeneous expression in a previous study^29^. In MOC1, CD34^+^ vessels of various sizes were distributed in the stroma area around the tumor nest. In MOC2, CD34^+^ vessels of various sizes were irregularly distributed within poor stromal areas (Figure 3A). CD105^+^ vessels were short, irregularly shaped, and expressed in the stromal area of MOC1. In MOC2, CD105^+^ vessels were short and irregularly expressed in poor stromal areas (Figure 3B). Micro-vessel density (MVD) indicates the extent of angiogenesis^30^. This correlates with the aggressiveness of different tumor types^31^,^32^. The MVDs of the CD34^+^ and CD105^+^ vessel structures were higher in the MOC2 group (Figure 3C, 3D). Hence, MOC1 is associated with low vascular density, whereas MOC2 exhibits high vascular density with irregularly distributed vessels in the poor stroma, suggesting a more aggressive angiogenic response. IHC analysis showed that CD34 and CD105 expression differed among the heterogeneous tumor vasculatures. To determine the characteristics of the vessel structures within the tumor stroma, we examined the lengths of CD34^+^ and CD105^+^ vessel structures in MOC1 and MOC2. CD34 is expressed in various-sized vessels in MOC1 and MOC2 tumors (Figure 3E). CD105 expression was associated with short-length vessels within MOC tumors (Figure 3F). The ratio of vessel length tended toward medium to long, ranging from 50-200≤ µm, within the CD34^+^ vessel landscape of MOC1 & MOC2 (Figure 3G, 3H). On the contrary, CD105^+^ vessel lengths trended toward shorter lengths, and most vessels are <50 µm in MOC1 & MOC2 (Figure 3I, 3J). The CD34^+^ vessels displayed a variety of sizes, indicating a mix of longer, stable, and actively forming vessels. In contrast, most vessels stained with CD105 were short, suggesting that they were new and actively forming blood vessels. This implies that the CD34^+^ and CD105^+^ vessels have different characteristics within the tumor stroma.

### CXCR4 is expressed on vessel-like structures in MOC1 and MOC2 tumor stroma

**Figure 4:**
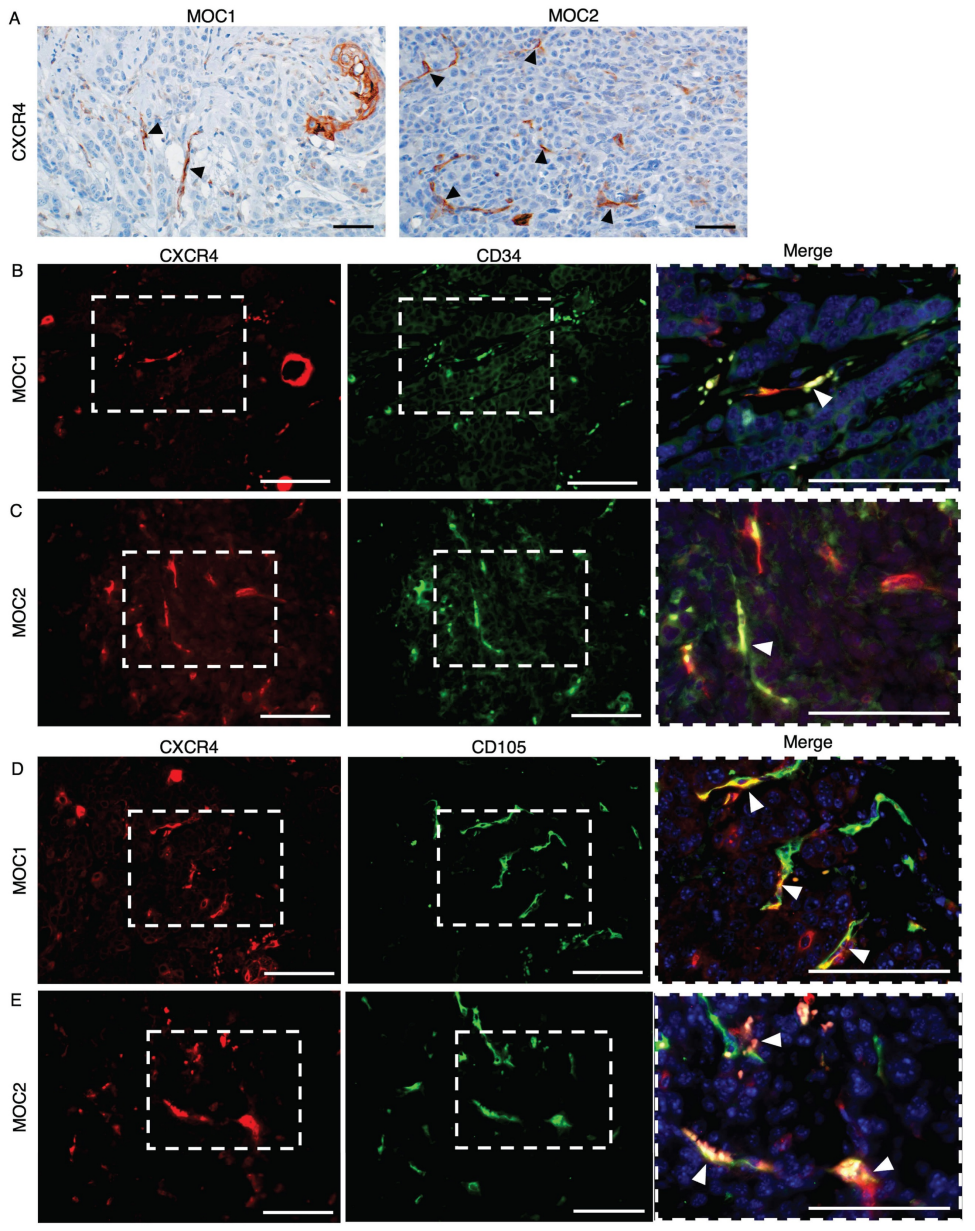
CXCR4 expression in MOC1 & MOC2 tumor tissues. IHC image analysis of CXCR4 shows positive expression on vessel-like structures of (A) MOC1 & MOC2. Arrows indicate positive vessel-like structures. Scale bar: 50 µm. IF image shows that CXCR4 expression is merged with CD34^+^ vessels in (B) MOC1 and (C) MOC2. CXCR4^+^ vessels are merged with CD105^+^ vessels in (D) MOC1 and (E) MOC2, indicating the involvement of CXCR4 in tumor vascularisation of both MOC tumors. Arrows indicate double-merged vessel structures. Scale bar: 50 µm.

To visualize the expression of CXCR4 in MOC1 and MOC2 tumor stroma, we performed IHC staining for CXCR4. CXCR4 is expressed in vessel-like structures and mononuclear cells, as well as in the keratinized area of MOC1. However, the expression of CXCR4^+^ vessel-like structures was weaker in MOC1 than in MOC2, and CXCR4 was abundantly expressed in the vessel-like structures within the tumor stroma (Figure 4A). To confirm that the CXCR4^+^ vessel-like structures were endothelial blood vessels, double immunofluorescence (IF) staining for the vascular markers CD34 and CD105 with CXCR4 was performed. CXCR4 expressions merged with CD34^+^ and CXCR4^+^ vessel-like structures in MOC tumors (Figure 4B-4E). These findings confirmed that CXCR4 is expressed in the blood vessel endothelial cells of MOC tumors and is involved in tumor vascularization.

### Consecutive IHC staining to detect co-localization of CXCR4 with endothelial markers CD105, CD34 and D2-40

**Figure 5:**
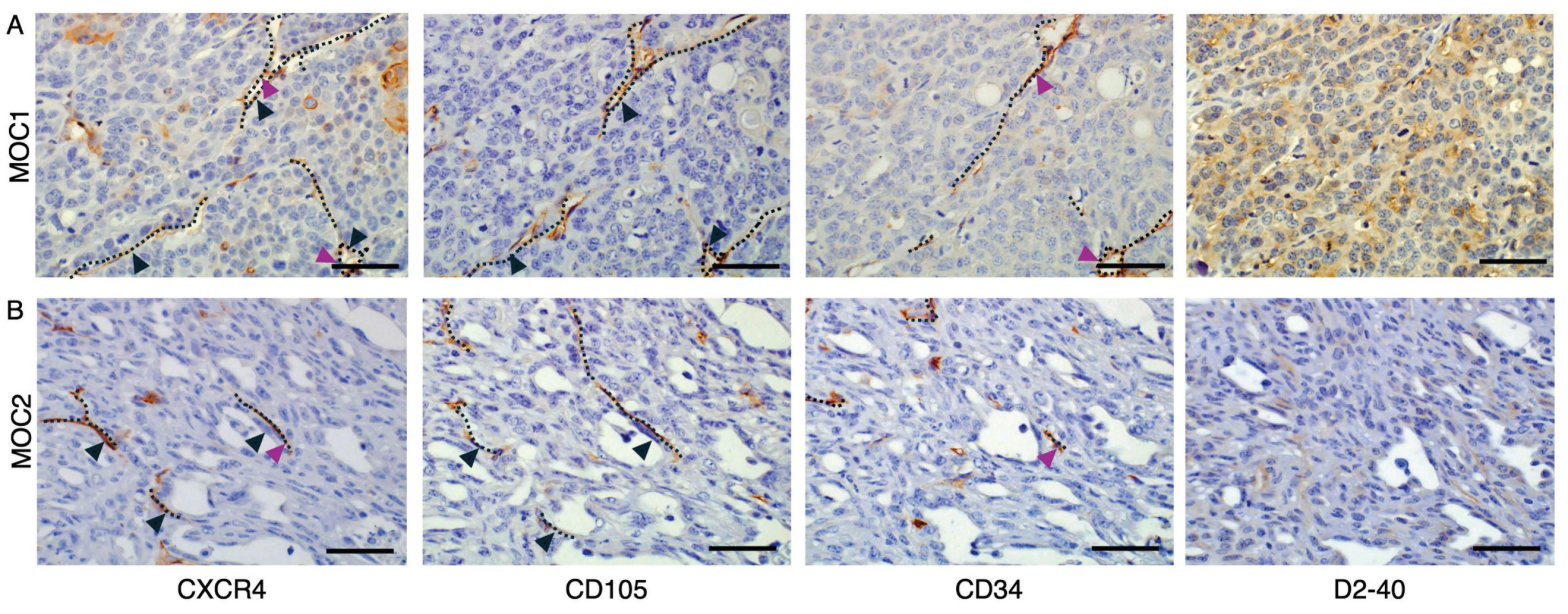
Detection of CXCR4 co-localization with endothelial markers using consecutive IHC stainings. In both MOC1 & MOC2 tumors, IHC analysis using serial consecutive sections revealed (A) In MOC1 tumor stroma, CXCR4^+^ vessel structures exhibit high overlap with CD105^+^ vessel structures. In contrast, co-localization with CD34^+^ vessels was limited, and D2-40^+^ lymphatic endothelial structures did not overlap with CXCR4^+^ vessels. (B) In MOC2 tumor stroma, CXCR4^+^ vessel structures also highly overlapped with CD105^+^ vessel structures and co-localization with CD34^+^ vessel was limited. D2-40^+^ lymphatic vessels do not overlap with CXCR4^+^ vessels. Dotted lines represent vascular structures. Black arrowheads represent CD105^+^ vessels overlapped with CXCR4^+^ vessel structures. Purple arrowheads represent CD34^+^ vessels overlapped with CXCR4^+^ vessel structures. Scale bar: 50 µm.

To detect the co-localization of CXCR4^+^ vascular structures with endothelial markers, we stained tumor tissues of both MOC1 and MOC2 in serial sections with CD105, CD34, and D2-40. In both MOC1 (Figure 5A) and MOC2 (Figure 5B), CXCR4^+^ vessel structures are shown to highly overlap with CD105^+^ vessel structures, but limitedly overlap with CD34^+^ vessel structures. These results indicate that CXCR4 is primarily expressed on the CD105^+^ active angiogenic vessels and is less involved with CD34^+^ mature vessel structures. D2-40 is widely used as a specific marker of lymphatic vessels 33,34, and these structures did not overlap with CXCR4^+^ vascular region. This suggests that CXCR4-expressing vessels are distinct from the lymphatic vasculature identified by D2-40.

### AMD3100 did not affect the proliferation of MOC cell lines but reduced the migration ability

**Figure 6:**
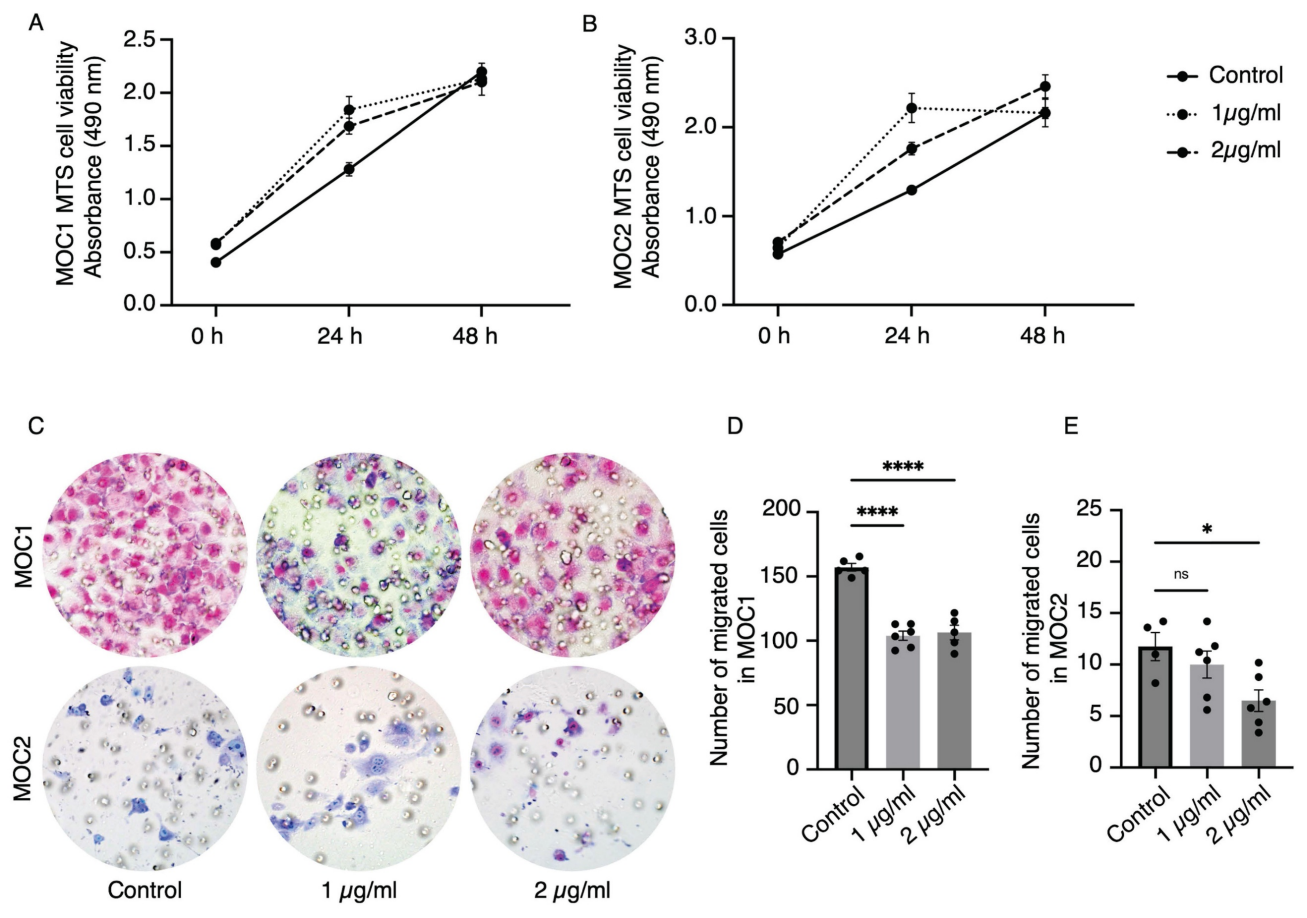
The effect of CXCR4 antagonist, AMD3100, on MOC1 and MOC2 cell lines in in vitro studies. MTS assays show no effect of AMD3100 on the proliferation of (A) MOC1 and (B) MOC2 cell lines. In the transwell migration assay (C), AMD3100 decreases the migration ability of both (D) MOC1 and (E) MOC2 cells. The experiments were repeated at least three times with consistent results, showing representative results. All data is presented as mean ± SD. Statistical analysis was done using one-way ANOVA followed by Tukey's multiple-comparison post hoc test. *P<0.1, ****P<0.0001.

To investigate the effect of AMD3100 on MOC1 and MOC2 cancer cells, a cell proliferation assay (MTS assay) and transwell migration assay were performed with two concentrations of AMD3100 used in previous studies by Uchida. at el^35^. The MOC1 and MOC2 drug-induced groups showed no reduction in proliferation compared to the control group at 24 and 48 h (Figure 6A, 6B). These results imply that the AMD3100 has no cytotoxic effect on MOC cancer cells and that CXCR4 antagonism does not directly affect the proliferation of cancer cells. In the Transwell migration assay, AMD3100 decreased the migration ability of MOC cells (Figure 6C). Both AMD3100 concentrations significantly affected the migratory ability of MOC1 cells (Figure 6D). MOC2 cells migration was significantly decreased with 2 µg/ml concentration (Figure 6E). These data suggest that CXCR4 influences the migratory abilities of MOC1 and MOC2 cancer cells.

### AMD3100 induced significant areas of tumor necrosis in poorly differentiated MOC2

**Figure 7:**
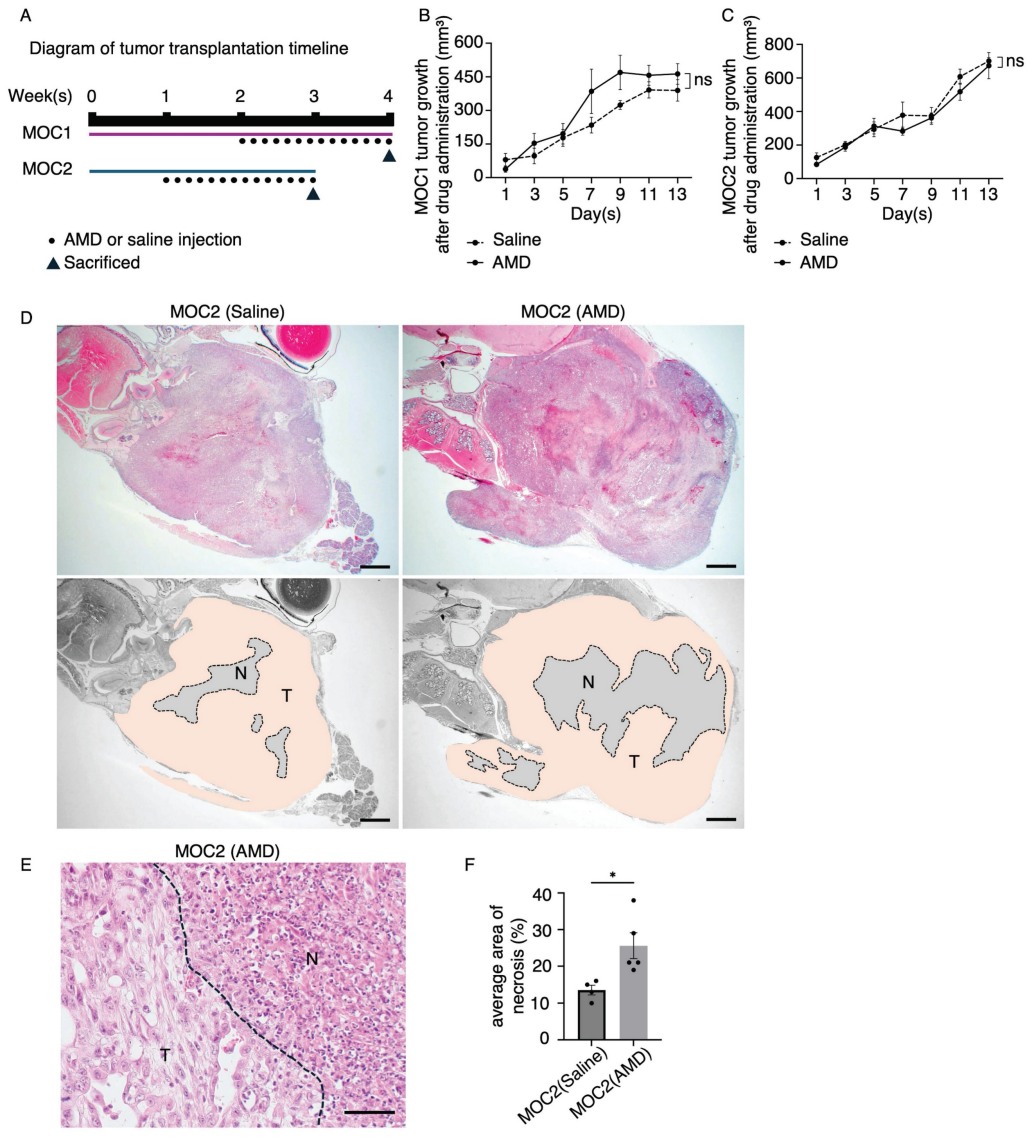
In vivo studies of the effect of AMD3100 in MOC1 and MOC2. (A) Illustration of tumor transplantation and AMD3100 or saline injection timeline. The average tumor volume response to drug treatment in the saline and AMD3100 groups shows no significant difference in (B) MOC1 and (C) MOC2. (D) MOC2 tumor shows a few necrosis areas observed in the saline group, and the AMD3100 group shows a large area of necrosis in the tumor tissue. T: tumor area. N: necrosis area. AMD: AMD3100. Scale bar: 1 mm. (E) The necrosis area has clear demarcation from tumour tissue with cellular debris. The dotted line represents the boundary of the tumor and necrosis area. T: tumor area. N: necrosis area. Scale bar: 50 µm. (F) The average area of necrosis is significantly increased in the drug-induced group. All data is presented as mean ± SD. Statistical analysis was done using the Student's t-test for comparison of two groups *P<0.1.

A schematic timeline of MOC tumor transplantation and saline or AMD3100 injection is shown (Figure 7A). The tumor growth response to drug treatment in MOC1 and MOC2 was not significantly different from that observed in the saline-treated group (Figure 7B, 7C). To investigate the effect of AMD3100 on the TME, we analyzed the H&E images of tumor tissues. The MOC1 tumors showed no histological differences between the saline and AMD3100 groups (Figure S1A). In MOC2 cells, H&E staining of the AMD3100-treated group revealed more abundant areas of necrosis across the tumor. The necrotic area was large, irregular, and located centrally within the tumor, with a bleeding area (Figure 7D). It was demarcated from viable tumor cells, suggesting ischemic necrosis with cellular swelling and nuclear dissolution (Figure 7E). The average area of necrosis was significantly increased in the AMD3100 group (Figure 7F). The increased necrosis and cell death in the AMD3100 group suggest that the vascular system supplying nutrients and oxygen for the survival of tumor cells may have been disrupted. This inadequate supply of oxygen and nutrients leads to hypoxia in the TME, thereby triggering necrosis.

### AMD3100 selectively impacted the micro-vessel density (MVD) of CXCR4^+^ and CD105^+^ vessel structures in MOC2

**Figure 8:**
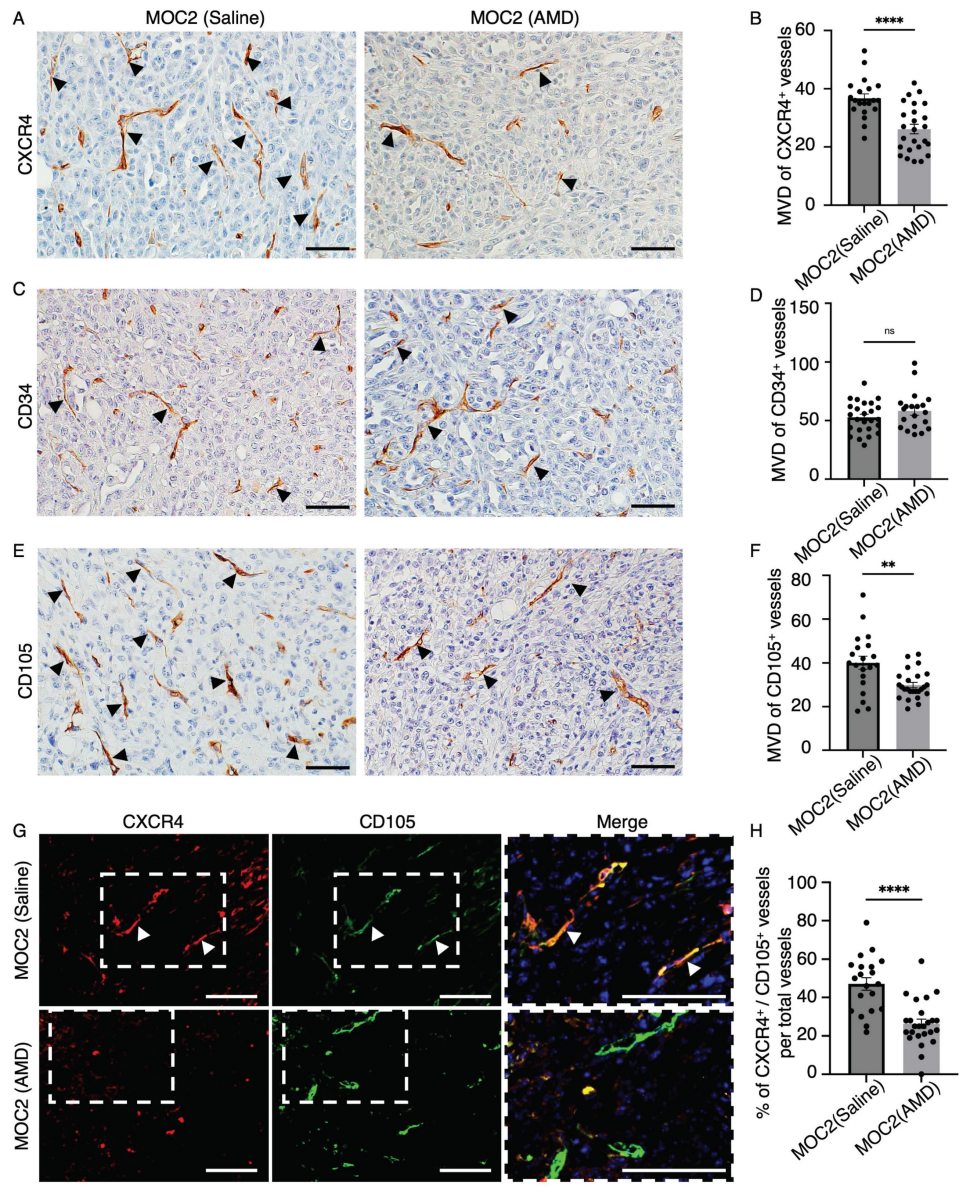
The effect of AMD3100 on the vascular density of MOC2. IHC images of (A) CXCR4^+^ vessel-like structures in the saline group and AMD3100 group. (B) The number of CXCR4^+^ vessel-like structures significantly decreases in the AMD3100 group. IHC images of (C) CD34^+^ vessel-like structures in the saline and AMD3100 groups. (D) The number of CD34^+^ vessel-like structures is not different in saline and the AMD3100 group. IHC images of (E) CD105^+^ vessel-like structures in the saline group and the AMD3100 group. (F) MVD of CD105^+^ vessel-like structures is significantly decreased in the AMD3100 group. Arrows indicate positive vessel-like structures. Scale bar: 50 µm. (G) IF images of CXCR4^+^/CD105^+^ merged vessel structures and (H) the percentage of merged vessels per total vessels is also significantly lower in the AMD3100 group. AMD: AMD3100. Arrows indicate double-merge vessel structures. Scale bar: 100 µm. All data is presented as mean ± SD. Statistical analysis was done using the student's t-test for comparison of two groups, **P<0.01, ****P<0.0001.

We want to investigate the effect of AMD3100 on CXCR4^+^ vessel structures in MOC tumors. Therefore, we examine the MVD of CXCR4^+^ vessel structures. In addition, we also counted the vessel structures marked by the vascular markers CD34 and CD105. Vascular density changes due to AMD3100 were not observed in the MOC1 vessel landscape, where the MVD was not different in CXCR4^+^ (Figure S2A, S2B), CD34^+^ (Figure S2C, S2D), and CD105^+^ vessels (Figure S2E, S2F). In MOC2, compared to the saline group, the AMD3100 group (Figure 8A) showed a decrease in the expression of CXCR4^+^ vessel structure, and MVD analysis revealed a significantly lower level of CXCR4^+^ vessel structure in the AMD3100 group (Figure 8B). We observed no differences in the MVD of CD34^+^ vessels between the control and AMD3100 groups (Figure 8C, 8D). The CD105^+^ vessel-like structures were more abundant in the saline group than in the AMD3100 group (Figure 8E). The MVD decreased significantly in the AMD3100 group (Figure 8F). Therefore, inhibition of CXCR4 affects the CXCR4^+^ and CD105^+^ vessels rather than CD34^+^ vessels. To specifically investigate whether the CXCR4 antagonism affects the MVD of the active angiogenic endothelial cells in MOC2, double IF of CXCR4 and CD105 was stained (Figure 8G). The percentage of CXCR4^+^/CD105^+^ merged vessel structures among all CD105^+^ vessels was counted, and these vessels were found to be significantly lower in the AMD3100 group (Figure 8H). These findings indicate that the inhibition of CXCR4 significantly diminishes the population of proliferating active angiogenic CD105^+^ vessels. The selective reduction in CD105^+^ vessel structures without changes in CD34^+^ vessel structures indicates that AMD3100 specifically targets proliferating angiogenic vasculature. This vascular disruption impairs blood supply, leading to necrosis, particularly in poorly differentiated tumors.

### AMD3100 also indirectly impacted the maturation of CD105^+^ vessels in MOC2

**Figure 9:**
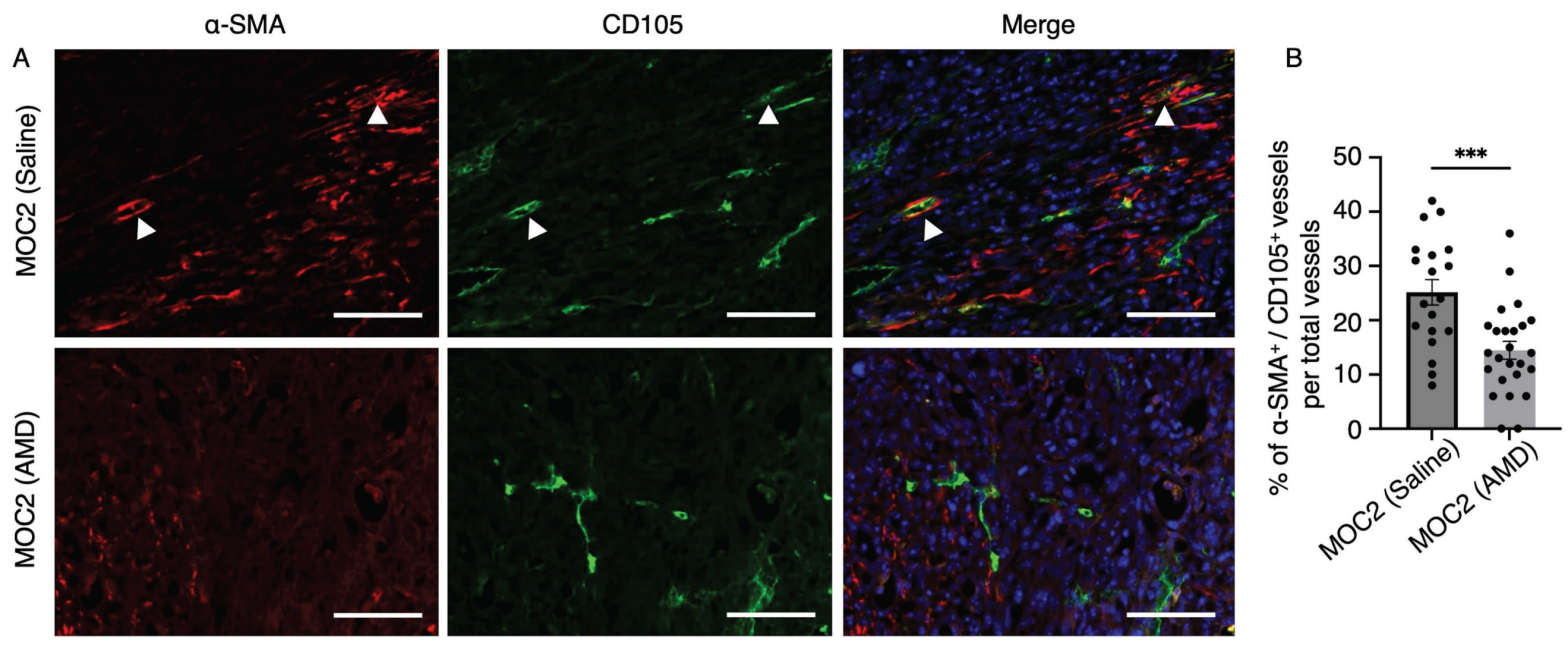
The effect of AMD3100 on the maturation of CD105^+^ tumor vessels in MOC2. (A) Double IF staining shows α-SMA^+^ pericytes closely associated with CD105^+^ vascular structures in the saline and AMD3100 groups. Arrows indicate α-SMA^+^ pericytes closely associated with CD105^+^ vessel structures. Scale bar: 100 µm. (B) Counting the percentage of double-positive vessel structures shows a significant decrease in the AMD3100 group compared to the saline group. AMD: AMD3100. All data is presented as mean ± SD. Statistical analysis was done using the Student's t-test for comparison of two groups ***P<0.001.

In addition to assessing the effect of AMD3100 on the vessel density of highly invasive MOC2 tumors, we also evaluated the levels of mature angiogenic vessels in the stroma. Alpha smooth muscle actin (α-SMA) is one of the common pericyte markers expressed on mural cells, which line the vessel structures for stabilization and support^36^,^37^. The α-SMA^+^ cells in close association with CD105^+^ micro-vessels were identified as pericytes, reflecting the active angiogenic state of the tumor vasculature, characterized by CD105 expression^38^. To investigate whether AMD3100 impacts the maturation of CD105^+^ angiogenic vessels, we investigated the IF images of α-SMA^+^/CD105^+^ vessels to compare the saline and AMD3100 groups (Figure 9A). The percentage of α-SMA^+^/CD105^+^ vessel structures among the overall CD105^+^ vessels was significantly lower in the AMD3100 group (Figure 9B). This result indicates that inhibition of CXCR4 reduces the recruitment of pericytes and indirectly disrupts the maturation of CD105^+^ vascular structures within the MOC2 stroma. The vessels become immature, leaky, and fragile, making them susceptible to rupture and regression, leading to necrosis.

## Discussion

In this study, we observed CXCR4-expressing vascular structures predominantly in higher pathological grades (AJCC grade 2 and grade 3) of human OSCC. The selective expression of CXCR4 on tumor-associated vessels in higher-grade OSCC suggests that CXCR4 may serve as a potential vascular biomarker, reflecting tumor progression and angiogenic activity. To further investigate the role of CXCR4 in tumor-associated vasculature, we utilized the murine oral cancer models. MOC1 and MOC2 are orthotopic mouse models derived from C57BL/6 wild-type mouse primary tumors developed from 7, 12-dimethylbenz (a) anthracene (DMBA)^39^. MOC1 is an indolent, well-differentiated phenotype derived from exophytic lesions, whereas MOC2 is aggressive and reflects invasive primary tumors with poor differentiation^39^. This study also noted that the highly invasive MOC2 is associated with a more significant number of vessel structures than MOC1. Thus, the MOC2 tumor variant with aggressive phenotypes necessitates an elevated density of blood vessels to compensate for rapid growth and metabolic demand^14^. The difference in vascular size indicates the heterogeneity of the vasculature morphology within the MOC tumor variants. The pan-endothelial marker CD34 is a cell surface sialomucin-like glycoprotein expressed by endothelial cells and is known to detect both newly formed and quiescent vessels^29^,^40^. The observation of CD34^+^ vessel structures of various sizes suggests its expression in a broader range of vessels, including larger, stable, mature vessels and shorter, tortuous, newly forming vessels. To selectively identify the active angiogenic vessels within the tumor stroma, CD105 was stained and visualized. The vascular marker CD105 (Endoglin) is a co-receptor of TGF-β, a pleiotropic cytokine that regulates cellular proliferation. CD105 is a proliferation-associated endothelial marker highly expressed in areas of vascular proliferation^38^,^41^. Consequently, vessel structures exhibiting positive staining for CD105 were characterized by their shortened and irregular morphology, highlighting their underdeveloped and newly formed nature.

We demonstrated the expression of CXCR4 in endothelial cells of stromal blood vessels in MOC1 and MOC2 tumors. Its co-localization with endothelial markers CD34 and CD105 further supports the role of the CXCR4 receptor in tumor vascularization and endothelial cell proliferation in MOC tumors. Although we did not quantify vessel co-localization, CXCR4 expression is observed more frequently in endothelial structures expressing CD105 than in those positive for CD34. This observation indicated a possible association between CXCR4 expression and activated or angiogenic vasculature. CXCR4 binding to SDF-1 promotes angiogenesis by activating PI3K/Akt and ERK/MAPK pathways, supporting endothelial cell survival, proliferation, and migration^22^,^25^,^26^. In various cancer models, including glioblastoma and HER-2 positive breast cancer, CXCR4 inhibition reduced tumor growth, invasion, metastasis, and stromal cell recruitment, highlighting its key role in tumor progression and the TME^25^,^42^,^43^. Several CXCR4 antagonists, especially AMD3100, have been investigated as therapeutic agents to disrupt its pathways and overcome resistance in various cancers^44^-^46^.

AMD3100, also known as plerixafor, acts as a specific antagonist of the binding pocket of CXCR4^47^,^48^. Initially developed as an anti-HIV drug, AMD3100 was later found to mobilize hematopoietic stem cells and progenitor cells from bone marrow to peripheral blood^49^. The U.S. Food and Drug Administration later approved AMD3100 for treating non-Hodgkin lymphoma and multiple myeloma^48^,^50^. While generally well-tolerated in clinical settings, potential side effects such as leukocytosis and adaptive resistance should be carefully considered^49^,^51^. Previous studies on inhibiting CXCR4 have primarily focused on metastasis and tumor invasion^23^,^35^,^52^, with limited investigation into its role in neovascularization, particularly in OSCC. Yoshida et al. discovered that AMD3100 induces tumor angiogenic inhibition-triggered necrosis (TAITN) in OSCC and enhances the efficacy of the chemotherapeutic drug, cisplatin^27^,^53^. This study employs a syngeneic mouse model with mouse cancer cell lines, allowing cancer and stromal cells to originate from the same host, thereby preserving natural TME interactions. We extensively explored the cause of the TAITN, focusing not only on vessel density expressed by CD34 and CD105 but also on the involvement of pericyte recruitment in an orthotopic mouse model.

The current study found no effect of AMD3100 on tumor cell proliferation in vitro. Hence, AMD3100 showed no direct cytotoxic effects on tumor cells. The response to AMD3100 in an in vivo study differed between the two MOC tumor variants. It is possible that AMD3100 does not affect MOC1 because of its low angiogenic profile or low dependence on CXCR4 for angiogenesis due to its well-organized stroma and less aggressive nature. In poorly differentiated MOC2, we observed the effect of AMD3100-induced ischemic-type necrosis and cell death. The discrepancy between the in vitro and in vivo effects of AMD3100 also highlights the influence of the TME. Although AMD3100 did not directly affect MOC1 and MOC2 proliferations in vitro, it disrupted the vascular system in the TME. This study highlighted the decline in CXCR4^+^ vessels along with CD105^+^ active angiogenic vessels, whereas the number of CD34^+^ vessels remained unchanged. This suggests that inhibition of CXCR4 selectively targets the active angiogenic CD105^+^ vessels rather than the broader CD34^+^ vasculature, which includes both stable and angiogenic vessels. AMD3100 possibly impairs the CXCR4-mediated recruitment and proliferation of CD105^+^ tumor vessels in the TME, leading to a hypoxic environment and triggering necrosis^42^,^54^,^55^**.** Therefore, CXCR4 is not only a marker of angiogenic vessels in TME but also functionally involved in tumor vascularization. The observed reduction in CXCR4^+^/CD105^+^ vessels following CXCR4 inhibition in the MOC2 model supports the therapeutic potential of targeting CXCR4 to disrupt tumor vasculature and limit tumor viability.

Angiogenesis not only depends on endothelial cell migration and proliferation but also requires coverage of mural cells for the maturation of newly formed vascular walls 36,56. Mural cells, especially pericytes, have been implicated as mediators of tumor angiogenesis^36^. Although the role of pericytes and their impact on anti-vascular drug resistance remains unclear, the benefit of targeting both endothelial cells and pericytes in anti-cancer therapies has been shown in several tumor models^37^,^57^. In this study, a reduction in α-SMA^+^ pericytes in close association with CD105^+^ vessels indicates that AMD3100 indirectly diminished the recruitment of the support cells required for the maturation of CD105^+^ angiogenic vessels, destabilizing the abnormal vessels. Therefore, inhibition of CXCR4 receptor may have therapeutic potential by selectively reducing angiogenic vascular density and delaying the maturation of CD105^+^ tumor micro-vessels essential for supplying the aggressive cancer phenotypes. An inadequate nutrient supply results in hypoxia within the TME, triggering necrosis. The findings in this study support the CXCR4 axis as a promising therapeutic target. CXCR4-directed treatments such as the antagonist AMD3100, could be particularly effective in patients with high-grade OSCC, where angiogenesis is more pronounced. In addition, this approach facilitates vascular normalization by inhibiting tumor micro-vessel development.

Tumor vascular normalization is the process of restoring disorganized tumor blood vessels to a more organized and functional state^58^. This approach has been shown to reduce tumor invasion and metastasis while enhancing the effectiveness of chemotherapy, radiation therapy, and immunotherapy^37^,^58^,^59^. CD34^+^ vessels, less affected by CXCR4 inhibition, may play a beneficial role in vascular normalization to improve the delivery of therapeutic drugs in combination with anticancer therapies. This finding opens avenues for incorporating CXCR4 inhibitors into personalized treatment regimens aimed at improving therapeutic outcomes. Future studies should explore CXCR4 inhibitors alongside anti-angiogenic, immunotherapeutic, or chemotherapeutic agents to suppress tumor progression. CXCR4 and VEGF signaling synergistically drive angiogenesis, and combination therapies may overcome VEGF-targeted resistance^26^,^55^.

The findings of our study conclusively demonstrate that the inhibition of CXCR4 induces necrosis through a reduction in the proliferation of angiogenic endothelial cells and hinders vessel maturation. Consequently, CXCR4 is a promising biomarker with significant potential for the treatment of poorly differentiated OSCC.

## Supplementary Material

Supplementary figures and table.

## Figures and Tables

**Table 1:** Association between CXCR4^+^vessel structures and clinical-pathological parameters in oral squamous cell carcinoma from tissue microarray of 59 patients

	Total n = 59	Number of cases with CXCR4^+^ vessel structure n = 25	Number of cases with CXCR4^-^ vessel structure n = 34	P value
Age				
< 60	40 (68%)	17 (68%)	23(68%)	
≥ 60	19 (32%)	8 (32%)	11(32%)	0.97
Sex				
M	50 (85%)	22 (88%)	28 (82%)	
F	9 (15%)	3 (12%)	6 (18%)	0.55
Differentiation				
High	33 (56%)	9 (36%)	24 (71%)	
Moderate	17 (29%)	10 (40%)	7 (21%)	
Poor	9 (15%)	6 (24%)	3 (9%)	**0.03**
Lesion site				
Tongue	28 (47%)	12 (48%)	16 (47%)	
Gingiva	14 (24%)	7 (28%)	7 (21%)	
Soft palate	2 (3%)	0 (0%)	2 (6%)	
Palate	1 (2%)	0 (0%)	1 (3%)	
Lip	2 (3%)	1 (4%)	1 (3%)	
Lower jaw	3 (5%)	2 (8%)	1 (3%)	
Maxillary sinus	2 (3%)	0 (0%)	2 (6%)	
Oral cavity	5 (8%)	3 (12%)	2 (6%)	
Cavioris bucca	2 (3%)	0 (0%)	2 (6%)	0.54
TMN staging				
Stage 1	5 (16%)	4 (3%)	1 (8%)	
Stage 2	38 (52%)	13 (74%)	25 (64%)	
Stage 3	15 (32%)	8 (21%)	7 (25%)	
Stage 4	1 (0%)	0 (3%)	1 (2%)	0.114

Bolded P value is significant by Chi-square test

## Abbreviations

CXCR4: C-X-C chemokine receptor type 4
TME: tumor microenvironment
OSCC: oral squamous cell carcinoma
VEGF: vascular endothelial growth factor
MOC: mouse oral cancer cell
IHC: immunohistochemistry
IF: immunofluorescence
H&E: hematoxylin and eosin
α-SMA: alpha smooth muscle actin
DMBA: dimethylbenz (a) anthracene
TGF-β: transforming growth factor beta
TAITN: tumor angiogenic inhibition-triggered necrosis
