# Supplementary material for: CXCR4 Inhibition Induces Tumor Necrosis by Selectively Targeting the Proliferating Blood Vessels in Oral Squamous Cell Carcinoma
Source: J Cancer. 2025 Sep 22;16(14):4055–70. doi: 10.7150/jca.115847 (PMC12595250; doi:10.7150/jca.115847)
Supplement: Supplementary file 1 — Supplementary figures and table. [file jcav16p4055s1.pdf]

Supplementary data

Figure S1

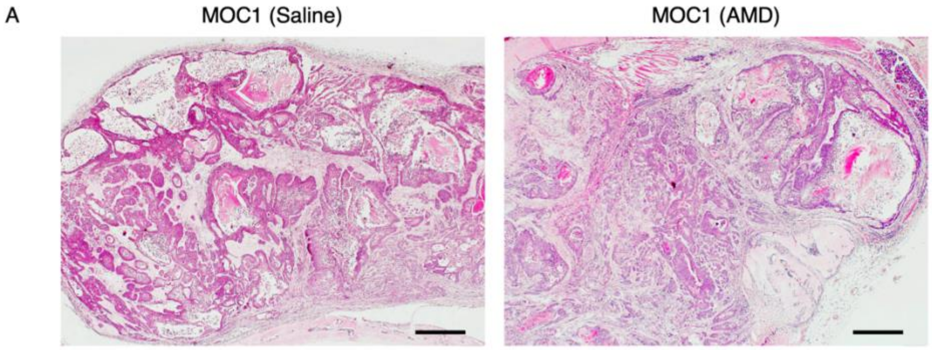

Figure S1. H&E analysis of MOC1 tumor. (A) The histological difference between the saline group and the AMD3100 group is not seen in MOC1 tumor phenotypes. Scale bar: 200 μm.

Figure S2

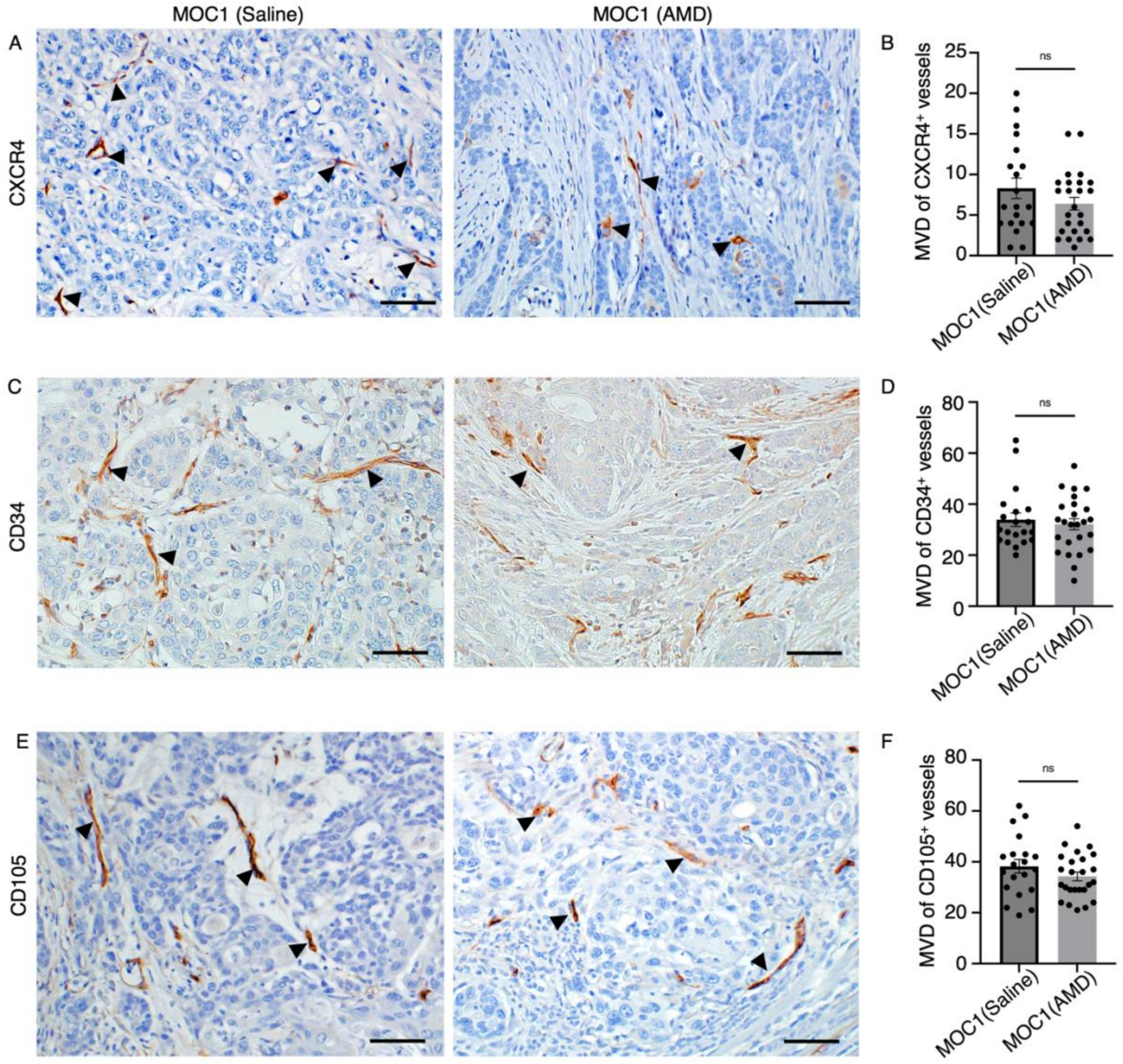

Figure S2. Analysis of micro-vessel density (MVD) in MOC1 tumour groups. IHC images of (A) CXCR4$^+$ vessel-like structure in saline and AMD3100 groups. (B) The number of CXCR4$^+$ vessel structures are not different in the saline and the AMD3100 group. IHC images of (C) CD34$^+$ vessel-like structures in the saline group and in the AMD3100 group. (D) The number of CD34$^+$ vessel-like structures are not different in the saline and the AMD3100 group. IHC images of (E) CD105$^+$ vessel-like structures in the saline and AMD3100 groups. (F) MVD is not different in the saline and the AMD3100 group. Arrows indicate positive vessel-like structures. AMD: AMD3100.

Scale bar: 50 µm. All data is presented as mean ± SD. Statistical analysis was done using the student's t-test for comparison of two groups.

Table S1. Summary of Antibodies details and usage

Primary Antibody	Immunized animal	Antigen Retrieval method	Dilution	Catalog number and Supplier
CXCR4	Rabbit	Pressure cooker heating in 0.01 mol/L citrate buffer (pH6.0) at 120°C for 1 min	1:300	ab124824 (Abcam)
CD34	Rat	Microwave heating in 0.01 mol/L citrate buffer (pH6.0) at 100°C for 1 min	1:500	ab8158 (Abcam)
CD105	Goat	Trypsin (0.1%), Calcium Chloride (0.1%) at 37°Cfor 5 min	1:80	AF1320 (R&D System Inc)
α-SMA	Rabbit	Microwave heating in 0.01 mol/L citrate buffer (pH6.0) at 100°C for 1 min	1:200	Ab5694 (Abcam)
D2-40	Rat	Microwave heating in 0.01 mol/L citrate buffer (pH6.0) at 100°C for 1 min	1:500	015-24111 (Wako)
